# The youngest male breast clear cell hidradenoma case: a literature review

**DOI:** 10.1186/s12887-026-06566-3

**Published:** 2026-03-13

**Authors:** Yavuz Yilmaz, Sukruye Demirkaya, Esra Karakus, Seda Sahin

**Affiliations:** 1 Department of Pediatric Surgery, Ankara Bilkent City Hospital, University of Health Sciences, Ankara, Turkey; 2 Department of Pediatric Surgery, Ankara Bilkent City Hospital, Ministry of Health, Ankara, Turkey; 3 Department of Pathology, Ankara Bilkent City Hospital, University of Health Sciences, Ankara, Turkey; 4 Department of Hematology, Yuksek Ihtisas University, Ankara, Turkey

**Keywords:** Clear cell hidradenoma, Pediatric breast tumor, Male breast lesion, Immunohistochemistry, p63, Diagnostic challenge

## Abstract

**Background:**

Clear cell hidradenoma (CCH) of the breast is an exceedingly rare benign adnexal tumor. Its clinical and radiological presentation can closely mimic malignancy, posing a significant diagnostic challenge.

**Case presentation:**

We report a case of a 6-year-old boy presenting with a non-healing, ulcerated nodule on the left nipple-areolar complex. Ultrasonography revealed a lobulated, cystic nodule. An ultrasound-guided core needle biopsy was performed, revealing epithelial cells with clear cytoplasm, warranting excision. The lesion was completely excised. Histopathology confirmed CCH, but an involved lateral margin necessitated a successful re-excision. Immunohistochemistry (IHC) on the excised specimen showed tumor cells positive for p63, Cytokeratin, and EMA, and negative for SMA, CD10, ER, and PR. The patient remains disease-free at 12 months.

**Conclusion:**

To our knowledge, this is the youngest male reported with breast CCH. This case highlights the diagnostic pitfall where CCH mimics malignancy and underscores the critical role of histopathology and IHC, particularly p63 positivity and myoepithelial marker negativity, in reaching an accurate diagnosis. Complete surgical excision with clear margins is the definitive treatment.

## Introduction

Clear cell hidradenoma (CCH) is a rare, benign adnexal tumor originating from the eccrine or apocrine sweat glands [1]. It typically presents as a solitary, slow-growing, dermal or subcutaneous nodule in the head, neck, and extremities of middle-aged adults [2]. Its occurrence in the breast tissue is uncommon, with a striking female predominance [3]. Diagnostically, CCH is challenging as it can mimic malignancy both on imaging (mammogram, ultrasound) and cytology (fine-needle aspiration), often leading to preoperative misdiagnosis [4, 5].

The pediatric breast presents a unique diagnostic challenge. While the vast majority of breast masses in children are benign, the clinical and radiological features of various lesions often overlap. This report details the case of a 6-year-old boy with CCH of the breast, who, to our knowledge, is the youngest male patient reported. We provide a detailed clinicopathological account emphasizing the diagnostic workflow, the pivotal role of immunohistochemistry (IHC), and discuss the management implications, including the importance of achieving clear surgical margins.

## Case presentation

**Fig. 1:**
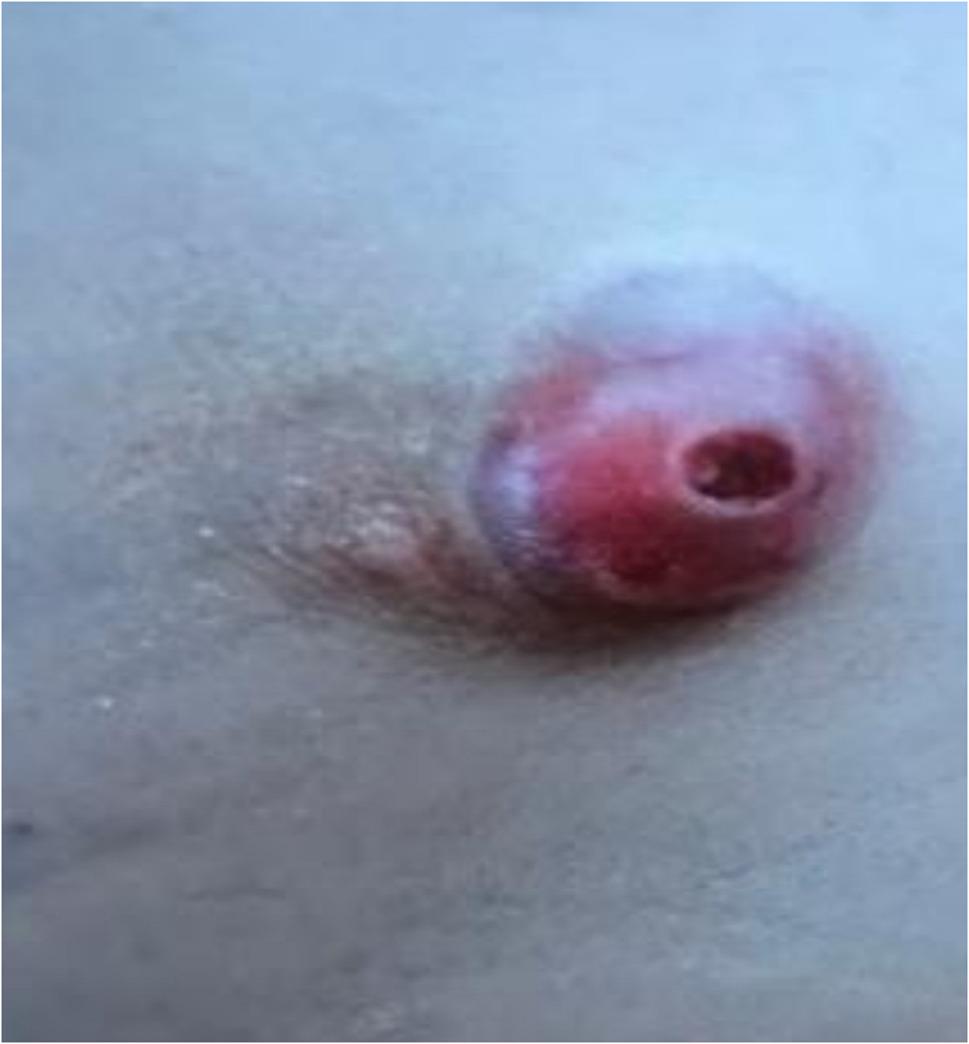
An ulcerated lesion on the superolateral aspect of the left breast

A previously healthy 6-year-old boy was referred with a several-month history of a non-healing, intermittently oozing wound on his left chest. Physical examination revealed a subcutaneous, firm, approximately 1-cm, ulcerated, and erythematous nodule superolateral to the left nipple-areolar complex (Fig. 1).

Breast ultrasonography revealed a well-defined, lobulated, hypoechoic, solid-cystic nodule (23 × 15 mm) in the subcutaneous plane with posterior acoustic enhancement. The radiological impression was suggestive of a benign adnexal tumor (e.g., epidermal inclusion cyst); however, a cystic papilloma or other neoplasms could not be entirely excluded. To obtain a preoperative diagnosis, an ultrasound-guided core needle biopsy was performed. Cytological examination revealed clusters of epithelial cells with clear cytoplasm, but a definitive diagnosis was not possible, prompting planning for diagnostic excision.

Under general anesthesia, the mass was completely excised using an ‘O-T plasty’ technique. Intraoperative frozen section was considered but not performed, as the initial plan was diagnostic excision with potential definitive treatment in one stage, depending on margins. Histopathological examination confirmed the diagnosis of clear cell hidradenoma but reported tumor involvement at the lateral deep surgical margin.

Given the known recurrence risk associated with incomplete excision, a shared decision was made with the family for re-excision. Two months later, a wide local re-excision of the scar bed was performed. Histopathology of the second specimen confirmed complete tumor removal with negative margins.

## Histopathological and immunohistochemical findings

### Gross and Microscopic Examination

**Fig. 2:**
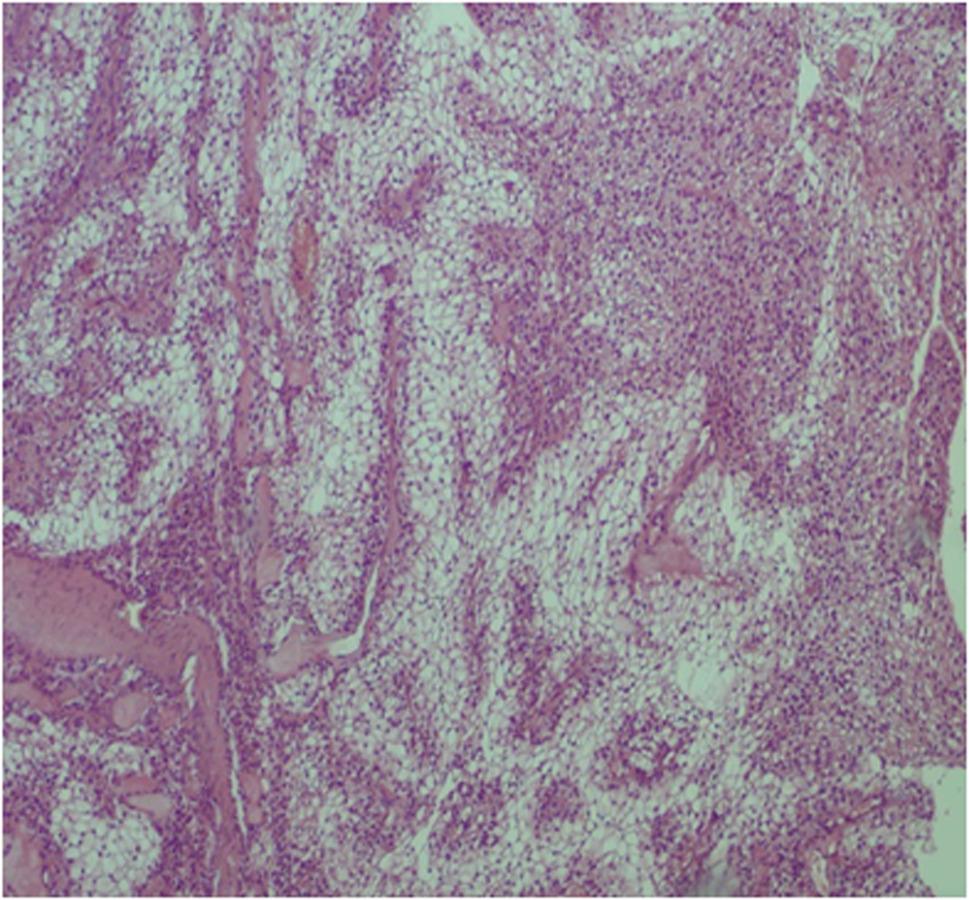
Haematoxylin and eosin stain at x10 magnification of the resected specimen revealing a lesion with polyhedral with a rounded nucleus and slightly basophilic cytoplasm nodules formed of sheets of uniform cells, and the cell nucleus appears small and dark

The initial specimen was a well-circumscribed, firm, grey-white dermal nodule. Microscopically, the tumor was unencapsulated but well-demarcated, situated in the dermis. It was composed of lobules and nests of two distinct cell types: polyhedral cells with eosinophilic cytoplasm and round cells with abundant clear, glycogen-rich cytoplasm, demonstrating PAS-positive, diastase-resistant staining, forming a classic biphasic pattern (Fig. 2A, B). Ductal differentiation was present. The tumor border was pushing rather than infiltrative. Tumor cells exhibited only mild nuclear atypia, and mitotic activity was virtually absent (< 1 mitosis per 10 high-power fields), effectively ruling out clear cell hidradenocarcinoma.

### Immunohistochemistry (IHC)

A comprehensive IHC panel was performed for definitive diagnosis and to rule out mimics (Table 1). The tumor cells showed strong positivity for epithelial markers (Cytokeratin AE1/AE3, EMA) and p63 (nuclear), confirming adnexal differentiation. Crucially, they were negative for myoepithelial markers (Smooth Muscle Actin - SMA, Calponin) and CD10. Hormone receptors (ER, PR) were also negative.

**Table 1:** Published male cases of clear cell hidradenoma of the breast

Author(s) and Year		Age (Years)	Key Demographic/Clinical Note
Finck et al. (1968) [6]		60	First reported male case in the literature
Kobayashi et al. (1994) [7]		63	
Domoto et al. (1998) [8]		44	
Shimizu et al. (1999) [9]		60	
Honnma et al. (2002) [10]		77	Oldest reported male case
Grampurohit et al. (2011) [11]		18	Previously reported youngest male case
Orsaria & Mariuzzi (2013) [12]		39	
Ogata et al. (2013) [13]		38	
Ano-Edward et al. (2018) [14]		62	
Current Case (2025)		6	Youngest male case in literature

## Discussion

### Demographic rarity and diagnostic challenge

Our case of a 6-year-old boy is the youngest male reported with breast CCH. This finding is particularly striking when viewed in the context of published series, which show an age range of 18–77 years with a strong female predominance [5]. A very recent case report in 2025 highlighted that only about seven cases involving male patients had been reported until then, underscoring the exceptional rarity of our pediatric male presentation [15]. While rare pediatric adnexal tumors exist, this case dramatically highlights that CCH can occur in early childhood and must be part of the differential diagnosis for pediatric breast nodules, regardless of gender [16]. The primary significance lies in illustrating the diagnostic pitfall: a lesion clinically presenting as an ulcerated mass and radiologically as a solid-cystic nodule, which can be easily misdiagnosed as malignancy or other benign tumors like intraductal papilloma [4, 5]. The literature indicates that preoperative misdiagnosis is common, with cases initially suspected to be carcinoma, leading to significant patient anxiety and potential for overtreatment [4, 16].

Our case of a 6-year-old boy extends this diagnostic challenge into early childhood and male gender, populations where CCH is scarcely considered. This presentation mandates that clinicians include CCH in the differential diagnosis of any pediatric breast nodule, particularly those with ulceration or a complex cystic appearance on ultrasound, to prevent misdiagnosis and guide appropriate, conservative surgical planning from the outset.

### Key diagnostic clues and the role of diagnostic tests

The diagnosis of CCH hinges on recognizing key histological and immunohistochemical clues [15]. Our case aligns with the classic description: a biphasic cellular morphology of clear glycogen-rich cells and eosinophilic cells, arranged in lobules separated by fibrovascular septa [5]. As emphasized in recent literature, diagnostic clues for CCH systematically include: (1) the presence of two distinct cell morphologies, (2) immunohistochemical staining indicating p63 positivity, (3) negativity for myoepithelial markers (e.g., SMA, calponin), and (4) MAML2 gene rearrangement in a significant subset of cases [15, 17].

The pivotal role of IHC is demonstrated in our case (Table 1). The profile of p63+/CK+/EMA+/SMA-/ER-/PR- is characteristic and crucial for distinguishing CCH from mimics [3, 5]. p63 positivity is a key diagnostic marker, often being the only myoepithelial marker expressed in CCH [3].

A critical diagnostic advancement is the identification of MAML2 gene rearrangement, resulting from a t(11;19) translocation. This molecular alteration is a defining feature in many CCHs and serves as a powerful diagnostic adjunct in challenging cases [15, 17, 18]. While not performed in our case, its utility is well-established.

Regarding biopsy, our approach differed from cases where diagnosis was achieved preoperatively via core needle biopsy (CNB) [5]. This highlights that CNB can be diagnostic when targeting the solid component, potentially aiding surgical planning. However, definitive diagnosis via fine-needle aspiration remains exceedingly difficult [2].

The diagnostic odyssey in our case—from non-healing ulcer to CNB with inconclusive cytology to definitive excision—highlights a practical clinical pathway. It reinforces that while CNB can be suggestive, as in our patient where clear cells prompted excision, the definitive diagnosis of CCH often rests on the histology and IHC profile of the completely excised specimen. Therefore, in similar pediatric presentations, a low threshold for diagnostic excision is justified, with the understanding that IHC (specifically for p63 and myoepithelial markers) will be the final arbiter.

### Surgical management and the importance of clear margins

The cornerstone of treatment is complete surgical excision with histologically clear margins. Our case perfectly illustrates the consequence of an involved margin and the necessity of re-excision. This aligns with literature reports indicating that incomplete excision is the primary risk factor for local recurrence [19–21]. This principle holds true regardless of patient age, as demonstrated in our pediatric case. This underscores that the surgical goal for suspected adnexal tumors should be complete excision with a cuff of normal tissue at the first attempt.

Our experience of requiring re-excision due to an initially involved margin serves as a critical learning point, especially in the pediatric breast where tissue preservation is paramount. It underscores that the goal of the first surgery should not merely be diagnostic, but curative. Achieving this may require a more generous initial excision margin than might be considered for other benign pediatric lesions, balancing oncologic safety with cosmetic outcome in this sensitive location.

## Conclusion

This report of the youngest male with breast CCH serves as a critical reminder of the diagnostic challenges posed by adnexal tumors in uncommon demographics. The case demonstrates that CCH can mimic malignancy clinically and radiologically, necessitating a systematic diagnostic approach. Accurate diagnosis integrates histology with a targeted IHC panel, where p63 positivity coupled with myoepithelial marker negativity is a cornerstone. For the pediatric surgeon, the paramount management principle remains complete surgical excision with clear margins to ensure cure. Awareness of this entity is essential to avoid diagnostic error, prevent unnecessary anxiety, and guide appropriate, definitive surgical intervention from the beginning.

## Data Availability

No datasets were generated or analysed during the current study.
